# Butyrylation of Maize and Potato Starches and Characterization of the Products by Nuclear Magnetic Resonance and In Vitro Fermentation

**DOI:** 10.3390/foods7050079

**Published:** 2018-05-18

**Authors:** Tina Skau Nielsen, Nuria Canibe, Flemming Hofmann Larsen

**Affiliations:** 1 Department of Animal Science, Aarhus University, Blichers Allé 20, DK-8830 Tjele, Denmark; Nuria.Canibe@anis.au.dk; 2 Department of Food Science, University of Copenhagen, Rolighedsvej 26, DK-1958 Frederiksberg C., Denmark; fhl@food.ku.dk

**Keywords:** butyrate, dietary fiber, resistant starch, esterification, NMR

## Abstract

Intake of butyrylated starches may increase colonic butyrate supply, which can be of public health and clinical benefit by maintaining colonic health. The objective was to investigate if an organocatalytic method with tartaric acid as a catalyst could be applied to produce butyrylated products from different starch sources and to characterize their chemical structure and fermentation capability by using solid-state ^13^C MAS NMR (magic angle spinning nuclear magnetic resonance) spectroscopy and an in vitro fermentation model, respectively. Low-amylose and high-amylose potato starch (LAPS and HAPS) and low-amylose and high-amylose maize starch (LAMS and HAMS) were subjected to organocatalytic butyrylation. This resulted in products with an increasing degree of substitution (DS) measured by heterogenous saponification and back titration with the HCl (chemical method) depending on reaction time. NMR analysis, however, showed that the major part of the acylation was induced by tartarate (75–89%) and only a minor part (11–25%) by butyrate. Generally, the chemical method overestimated the DS by 38% to 91% compared with the DS determination by NMR. Increasing the DS appeared to lower the in vitro fermentation capability of starches independent of the starch source and, therefore, do not seem to present a feasible method to deliver more butyrate to the colon than lower DS products.

## 1. Introduction

The short chain fatty acids (SCFA) acetate, propionate, and butyrate produced by microbial fermentation of dietary fiber (DF) in the large intestine are believed to play a beneficial role in gut health [1]. In particular, butyrate appears to be important for maintaining metabolic homeostasis in colonocytes. Apart from being the preferred energy source for colonic epithelial cells [2] and a major regulator of cell proliferation and differentiation [3], butyrate has been shown to possess anti-carcinogenic [4], anti-inflammatory, and anti-oxidant [5] effects in in vitro and animal studies and overall to enhance the intestinal barrier function and mucosal immunity [6]. Many of these effects have been related to its action as a histone deacetylase inhibitor [7].

Strategies to increase the production of specific SCFA’s in the colon may be of public health and clinical benefit. One way to achieve this is to increase the consumption of foods high in resistant starch (RS) because RS fermentation generally favors butyrate production in the large intestine [8,9]. However, there is considerable variation among individuals regarding the ability to ferment RS and it appears that the microbiota of some humans [10] and pigs [11] cannot ferment certain types of RS. Another strategy is to acylate starches with specific SCFA in which the acyl group is linked to the starch framework by an ester bond. Acylation of starches has been shown to be an effective means of delivering specific SCFA to the colon of humans [12,13] and animals [14] because they are more resistant to small intestinal amylolysis and are delivered to the colon for release by bacterial esterases. Butyrylated high-amylose maize starch (HAMSB) was shown to protect the colonic mucosa of rats from DNA damage induced by a high-dietary protein diet [15] to reduce colorectal cancer markers dose-dependently in the azoxymethane (AOM) induced rat model of colorectal-cancer [16] as well as to prevent red-meat induced promutagenic DNA-adducts in the rectal tissue of humans [17]. The HAMSB applied in these studies had a degree of substitution (DS) of approximately 0.25. This means that 8% of the hydroxyl groups on each d-glucopyranosyl unit in starch is esterified with butyric acid.

Most commercial starch esters are produced by a reaction of starch in aqueous medium with anhydrides and NaOH as catalyst. However, Tupa et al. [18] described an operationally simple and more environmentally-friendly laboratory scale method where native low-amylose maize starch was butyrylated using a carboxylic acid (butyric acid) with tartaric acid as the non-toxic green catalyst in the absence of solvents. The DS, which is determined by heterogenous saponification and back titration with HCl, was shown to reach approximately 1.5 after 5 h of reaction time at 120 °C. This is substantially more butyrate attached to the starch polymer compared to the DS = 0.25 HAMSB applied in previous animal and human studies [14,15,16,17]. Some studies indicate that DS values of particular maize starches under the same reaction conditions depend on the amylose-amylopectin ratio of the starch [19].

Therefore, the aim of this study was to examine if the methodology described by Tupa et al. [18] could be used to produce high DS butyrylated starches from different starch sources (maize and potato) with varying proportions of amylose (low vs. high amylose). It was also the aim to characterize these butyrylated starches in terms of chemical structure and fermentation capability by using solid-state ^13^C MAS NMR spectroscopy and an in vitro fermentation model system, respectively.

## 2. Materials and Methods

### 2.1. Starches

The four starches subjected to butyrylation were pre-gelatinized low-amylose maize starch (LAMS, C gel instant 12018, Cargill Nordic A/S, Charlottenlund, Denmark), high-amylose maize starch (HAMS, Hi-Maize^®^ 260, Ingredion, Bridgewater, NJ, USA), pre-gelatinized low-amylose potato starch (LAPS, ColdSwell 1111 Series, KMC, Brande, Denmark), and high-amylose potato starch (HAPS, not commercially available, KMC, Brande, Denmark). The HAMS and HAPS are products of traditional plant breeding (hybrids), i.e., selection over generations and not derived from genetically modified plants. Dry matter (DM) content was determined by drying at 103 °C to constant weight and ash was analyzed according to the AOAC (Association of Official Analytical Chemists) method (923.03) [20]. Total starch was determined by an in vitro starch digestion assay described by Englyst [21] and RS by a commercially available kit (Megazyme International, Wicklow, Ireland). The amylose/amylopectin content was also analyzed by a commercially available kit (Megazyme International, Wicklow, Ireland), according to the manufacturer’s instructions.

### 2.2. Organocatalytic Butyrylation of Starches

Esterification of LAMS, HAMS, LAPS, and HAPS with butyric acid was performed on a small scale in oven-dried 20 mL glass vials sealed with screw caps, according to a simple organocatalytic method described by Tupa et al. [18]. Butyric acid (10 mL, ≥99%, Sigma Aldrich, Brøndby, Denmark), l-(+)-tartaric acid (catalyst, 1.48 g, ≥ 99.5%, Sigma Aldrich, Brøndby, Denmark) and oven-dried starch (800 mg) were mixed in a glass vial and heated to 120 °C in a heat block with constant magnetic stirring. When the mixture achieved the target temperature, all tartaric acid dissolved and this was considered the beginning of the reaction. Esterification was run for different reaction times (0.5 h, 1 h, 1.5 h, 2 h, 2.5 h, 3 h, and 5 h) as well as 0.5–2.5 h of reaction times in triplicate (missing data for HAPS at 2.5 h of esterification, no replicates for LAPS at 2.5 h of esterification) and no replicates at 3 and 5 h of esterification. After the chosen reaction time, the mixture was allowed to cool to room temperature and the product was washed several times with distilled water by vacuum filtration in a Buchner funnel to guarantee the removal of the catalyst and unreacted acid. The solid was oven dried overnight at 40 °C to 50 °C.

### 2.3. DS Determined by Heterogeneous Saponification and Back Titration

Determination of the acyl content and degree of substitution (DS) was performed by heterogeneous saponification and back titration with HCl, which was described by Tupa et al. [18]. The acyl content was calculated by Equation (1), which is shown below.

(1) $$\mathrm{acyl}\;(\%)\;=\;\frac{(V_{B}-V_{S})\;\times \;N_{\mathrm{HCl}}\;\times \;M_{\mathrm{acyl}}\;\times \;10^{-3}\;\times \;100\%}{W}$$

where *V_B_* = volume (mL) HCl required for titration of the blank (= non-treated starch), *V_S_* = volume (mL) HCl required for titration the sample, *N*_HCl_ = normality of the HCl solution (= 0.1 M), *M*_acyl_ = molecular weight of the acyl group (71 g/mol for butyryl), and *W* = mass of sample used (g).

The degree of substitution (DS) of an acylated starch is defined as the number of hydroxyl (OH) groups substituted by an acyl group per anhydroglucose unit of the starch polymer. Since the anhydroglucose unit possesses three hydroxyl groups at carbon 2, 3, and 6, the maximum DS value is 3. When DS = 0.25 for a butyrylated starch, ~8.3% of the hydroxyl groups on each d-glucopyranosyl unit in starch is esterified with butyric acid.

The DS of acylated starches was calculated by using Equation (2) [18] assuming all acyl groups originate from butyryl.

(2) $$\mathrm{DS}\;=\;\frac{162\;g/\mathrm{mol}\;\times \;\mathrm{acyl}\;\%}{M_{\mathrm{acyl}\;}\times \;100\%\;-((M_{\mathrm{acyl}}-\;1g/\mathrm{mol})\times \;\mathrm{acyl}\;\%)}$$

where 162 g/mol is the molecular weight of the anhydroglucose units.

### 2.4. Characterization by Solid-State ^13^C MAS NMR Spectroscopy

Non-treated LAMS, HAMS, LAPS, and HAPS (not subjected to the organocatalytic esterification process) and butyrylated LAMS, HAMS, LAPS, and HAPS at a low and high DS determined by the chemical method were subjected to solid-state ^13^C MAS NMR spectroscopy analysis. All solid-state NMR spectroscopic experiments were carried out using a Bruker Avance 400 (9.4 T) NMR spectrometer (Rheinstetten, Germany) operating at Larmor frequencies of 400.13 MHz and 100.63 MHz for ^1^H and ^13^C, respectively. ^13^C single-pulse (SP) MAS and cross-polarization (CP) MAS [22] NMR experiments were recorded at 294 K using a double-tuned CP/MAS probe equipped for 4 mm rotors employing a spin-rate of 9 kHz, RF (radio frequency)-field strengths of 70 kHz for both ^1^H and ^13^C, 600 scans, a spectral width of 50.13 kHz, and an acquisition time of 40.9 ms during which TPPM (two pulse phase modulation) [23] ^1^H decoupling was applied. For the ^13^C CP/MAS experiments a contact time of 1.0 ms and a recycle delay of 16 s was utilized, a recycle delay of 128 s was used for the ^13^C SP/MAS experiments. Prior to Fourier Transformation, all FID (free induction decay)’s were apodized by a Lorentzian line-broadening of 10 Hz. All spectra were referenced to the carbonyl resonance of α-glycine at 176.5 ppm (external sample).

Determination of the relative ratios of glucose (starch), butyrate, and tartarate were obtained by integrating the region of the two aliphatic carbons not attached to the carbonyl in butyrate (5–25 ppm), the region of the anomeric carbon (C1) in starch (90–120 ppm), and the carbonyl carbon in the acid/ester (155–190 ppm). Due to the different longitudinal relaxation times for CH (hydrogen bearing carbon)’s and carbonyls, the ratio between C1 and the carbonyls was determined from the ^13^C SP/MAS spectra. The ratio between the aliphatic carbons in butyrate and C1 was determined from the ^13^C CP/MAS spectra due to a higher S/N-ratio. Before calculating the ratios, the integrals were normalized according to the number of carbon sites contributing to the integrals.

### 2.5. In Vitro Fermentation

In vitro 24-hour batch fermentations using human fecal inoculum were conducted on the four non-treated starches and on four selected butyrylated starches with increasing DS from each starch source to determine if the DS measured by heterogenous saponification and back titration affected the fermentation pattern in vitro. The method described by Edwards et al. [24] was modified and applied using a fecal inoculum, which was produced by combining 25 g samples of fresh fecal material from two volunteers who had not received antibiotics or laxatives and had not suffered from gastro-intestinal infections for at least two months prior to the study. Each fecal sample was collected and processed within 12 h. One hundred mL of 0.1 M anaerobic sodium phosphate buffer (pH 6.5) was added to the fecal inoculum (50 g) in a stomacher bag (33.3% weight/weight fecal slurry) with separator and flushed with N_2_. Following 6 min of stomaching, the supernatant was filtered into a sterile bottle while flushing with N_2_. Triplicates of 100 mg starch samples were weighed and placed into Hungate tubes. The samples were dissolved in 7 mL sterile 0.1 M sodium phosphate buffer (pH 6.5). Three mL of the fecal inoculum slurry was added to the tubes, which resulted in 10% (weight/weight) fecal inoculum and 1% (weight/weight) substrate and flushed with N_2_. A positive control (100 mg inulin + 7 mL sodium phosphate + 3 mL fecal slurry) and a negative control (7 mL sodium phosphate (pH 6.5) + 3 mL fecal slurry) were also included in the experiment. Tubes were placed horizontally in an incubator shaker and incubated at 37 °C for 24 h. Samples for SCFA determination were obtained at time 0 h and 24 h and stored at –18 °C until further analyzed.

Organic acid concentration was analyzed by gas chromatography (HP-6890 Series Gas Chromatography, Hewlett Packard, Palo Alto, CA, USA) using an SGE-BP1 column (30 m × 0.25 mm × 0.25 µm, Trajan Scientific, Ringwood, Australia) with 5% phenylpolysiloxane and 95% dimethylpolysiloxane and a flame ionization detector after submitting the samples to an acid-base treatment followed by ether extraction and derivatization, which was described by Canibe et al. [25].

### 2.6. Statistical Analysis

One-way ANOVA was conducted to estimate the impact of butyrylation on butyric acid and total organic acid concentration following the in vitro fermentation using SAS (SAS Institute, Cary, NC, USA). The Tukey-Kramer *post hoc* test was applied to adjust for multiple comparisons.

## 3. Results

### 3.1. Starch Composition

As expected, the content of amylose was 2–2.6 times higher in the HAMS and HAPS compared to the low-amylose starches with HAMS having the largest proportion of amylose (79.5% of dry matter, Table 1). The content of amylose in the HAMS and HAPS determined by the Megazyme kit corresponded well with the manufacturer´s declared content. Therefore, we did not find it reasonable to doubt the accuracy of the Megazyme kit measurements on the HAMS and HAPS nor find it necessary to conduct analysis of the molecular properties of the amylopection fraction of these high-amylose starches. The low-amylose starches contained almost no RS while the RS content of high-amylose starches was substantial (45% and 29% of total starch for HAMS and HAPS, respectively).

### 3.2. Esterification of Starches

**Figure 1:**
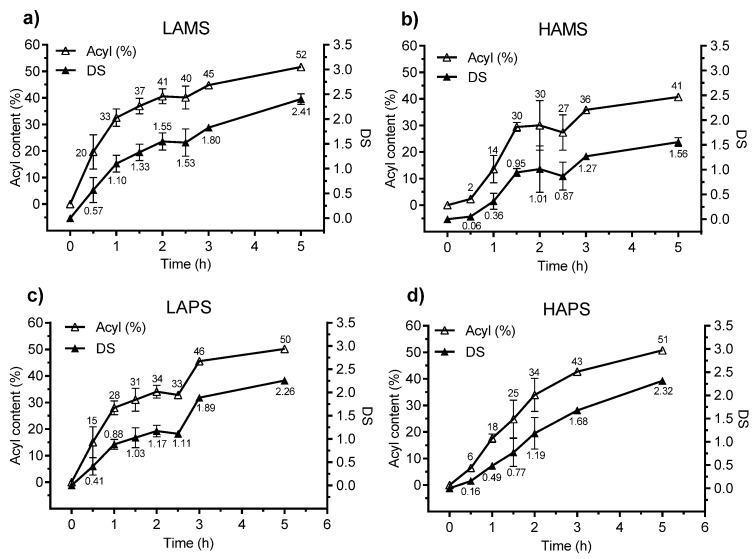
Effect of reaction time on the organocatalytic butyrylation of (**a**) low-amylose maize starch (LAMS), (**b**) high-amylose maize starch (HAMS), (**c**) low-amylose potato starch (LAPS), (**d**) high-amylose potato starch (HAPS). DS: degree of substitution.

Figure 1 illustrates the development in esterification of the four starches over the reaction time at a constant (120 °C) temperature. The LAMS (see Figure 1a) showed a higher reaction rate than HAMS (see Figure 1b) and already after 1 h a DS = 1.10 was achieved. The DS continued to increase over time for LAMS without reaching a plateau and the final DS value was 2.41 after 5 h of reaction compared with 1.56 for the HAMS.

The LAPS (see Figure 1c) also showed a higher reaction rate than the HAPS (see Figure 1d) up till 1.5 h (DS of 1.03 vs. 0.77 for LAPS and HAPS, respectively) but, after 5 h of reaction, the final DS for the two potato starches were quite similar. Overall, the LAMS, LAPS, and HAPS were esterified to a higher DS after 5 h than the HAMS.

### 3.3. Characterization of Starches by NMR Spectroscopy

As seen in Table 2, the DS of the eight butyrylated starches selected for analysis with either low or high DS based on the chemical method, which differed from the DS determined by solid-state ^13^C MAS NMR spectroscopy. The most striking difference between the two methods is that solid-state NMR analysis allows for discrimination between acylation by tartarate and butyrate. Therefore, it was shown that the major part of the acylation was induced by tartarate (75–89%) and only a minor part (11–25%) by butyrate. However, tartaric acid is a dicarboxylic acid and both of the acid groups may in principle react with a hydroxyl group on a glucose residue in starch. This makes DS with tartarate a more difficult parameter to handle and it is, therefore, more correct to provide the DS as the molar ratio between the acid and the anhydroglucose units, which is also the original output of the NMR-analysis (see Table 2). For both acids, a longer reaction time provided a higher degree of acylation. In general, the chemical method overestimated the DS 38 to 91% compared with the DS determination by NMR except for HAMS-low DS (see Table 2).

**Figure 2:**
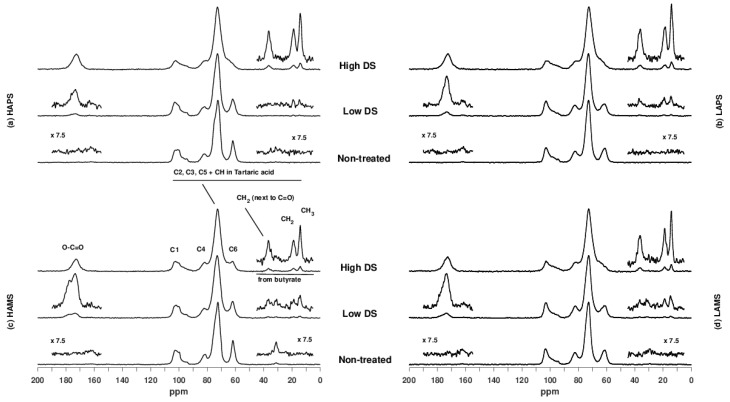
^13^C CP/MAS NMR spectra of (**a**) high-amylose potato starch (HAPS), (**b**) low amylose potato starch (LAPS), (**c**) high-amylose maize starch (HAMS) and (**d**) low-amylose maize starch (LAMS) either as non-treated starches or with low or high degree of substitution (DS).

Figure 2 shows the ^13^C CP/MAS NMR spectra of the different starches. The spectrum of high-DS HAMS contains assignments of the six carbons (C1–C6) of the α-1,4 linked glucose units as well as the carbons originating from tartaric and buryric acid. Comparison of the four non-treated starch samples shows marked differences in the distribution of C1 resonances as well as the line width of the C6 resonance for the high and low amylose samples due to the pre-gelatinization of the latter. Furthermore, observation of lipid (~32 ppm) in the HAMS sample indicates the presence of lipid-amylose complexes in this sample. Apart from the observations on the non-treated starch samples, a significant decrease in the intensity of the C6 resonance as a function of the DS was observed for all samples. This demonstrates that the acylation takes place on C6, which was expected. Because of the spectral overlap of resonances from C2, C3, and C5 in starch and CH in tartaric acid, it was not possible to verify if acylation also takes place on C2 and C3.

### 3.4. In Vitro Fermentation of Esterified Starches

**Figure 3:**
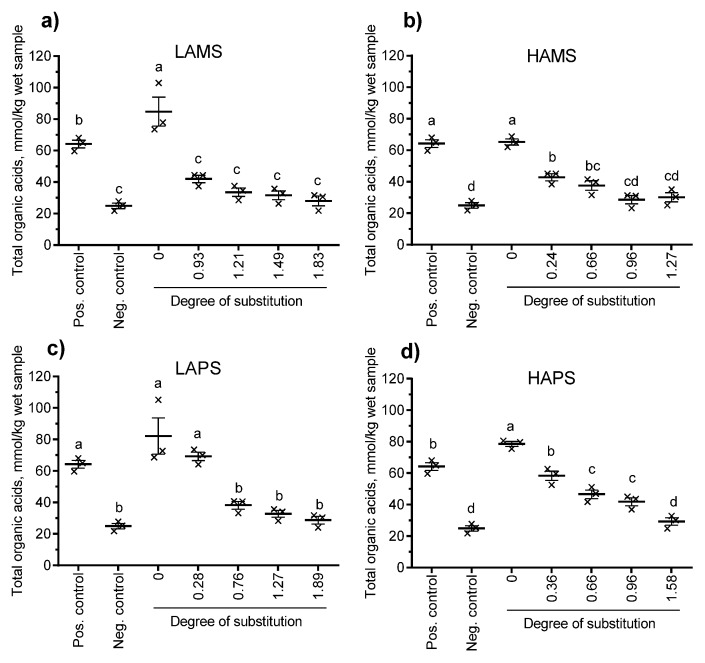
Effect of degree of substitution determined by the chemical method of (**a**) low-amylose and (**b**) high-amylose maize starch and (**c**) low-amylose and (**d**) high-amylose potato starch on the in vitro production of total organic acids after 24 h of incubation with human fecal inoculum. Data points with different letters are significantly (*p* ≤ 0.05) different from one another. Pos. control: positive control, Neg. control: negative control.

**Figure 4:**
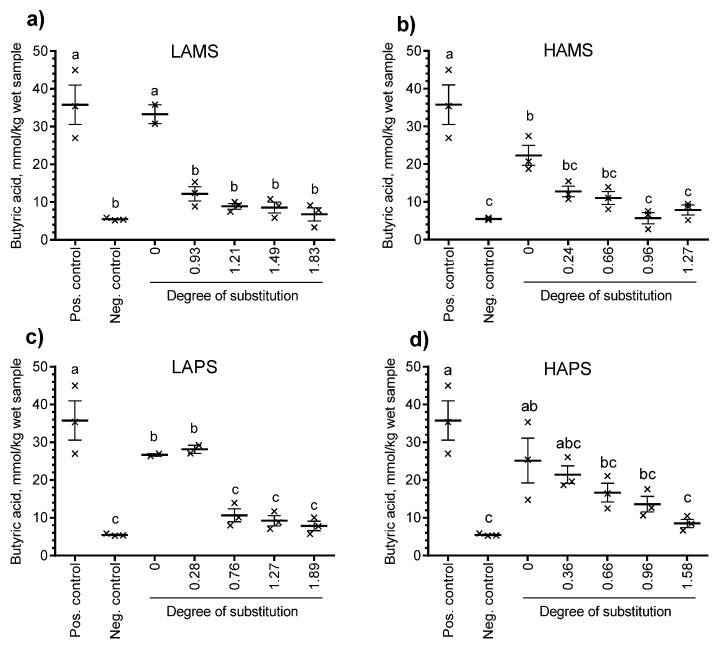
Effect of degree of substitution determined by the chemical method of (**a**) low-amylose and (**b**) high-amylose maize starch and (**c**) low-amylose and (**d**) high-amylose potato starch on the in vitro production of butyric acid after 24 h of incubation with human fecal inoculum. Data points with different letters are significantly (*p* ≤ 0.05) different from one another.

Figure 3 shows the production of total organic acids (mmol/kg wet sample) and Figure 4 shows the production of butyric acid from baseline corrected samples at 24 h. Besides the non-treated starches (DS = 0), esterified starches at four different levels of DS determined by the chemical method for each type of starch were included. All four non-treated starches resulted in a total organic acid production ranging from 65.2 mmol/kg to 84.8 mmol/kg wet sample, which was similar or higher than the positive control (inulin, 64.2 mmol/kg wet sample). Only LAPS esterified to DS = 0.28, HAMS esterified to DS = 0.24 and 0.66 and HAPS esterified to DS = 0.36, 0.66, and 0.96 resulted in significantly higher total organic acids than the negative control (no starch, 24.9 mmol/kg wet sample). The general trend following in vitro fermentation of butyrylated starches from the four sources increased DS and reduced the production of total organic acids (sum of formic, acetic, propionic, iso-butyric, butyric, iso-valeric, valeric, iso-capronic, capronic, heptanoic, sorbic, benzoic, lactic, and succinic acid). A similar trend was observed for butyrate. Only LAPS butyrylated to DS = 0.28 showed a butyrate production significantly higher (28.1 mmol/kg wet sample) than the negative control (5.5 mmol/kg wet sample). When the butyrate: total organic acid ratio was calculated (data not shown), there was no significant difference between the esterified starches and the non-treated starch for any of the four starch sources.

## 4. Discussion

The aim of this study was first to examine if the organocatalytic methodology for butyrylation of starches [18] could be applied to produce high DS butyrylated starches from different sources (maize and potato) with varying proportions of amylose. Second, the aim was to characterize the products in terms of chemical structure by NMR spectroscopy and their in vitro fermentation in order to examine if higher DS results in more butyrate are potentially delivered to the colon.

When attempting to develop starch products acylated with SCFA [18,19,27] that have beneficial effects in relation to gut health upon ingestion, maize starches have so far gained the most attention [15,16,17]. Our results show that, depending on reaction time, low-amylse and high-amylose potato starch can also be esterified to a similar or higher DS than the maize starches when applying an organocatalytic route of synthesis [18]. This indicates that potato starches have a potential as raw materials in the production of butyrylated starches for possible gut health promoting purposes. They even seem more efficient at holding butyrate than the maize starches after 5 h of reaction since the DS increased to an average of 0.29 for the LAPS and HAPS compared with 0.17 for LAMS and HAMS determined by ^13^C MAS NMR. Maize and potato starch differ in crystallinity (type A for maize and type B for potato) but there are many other morphological, structural, and molecular differences between the two types of starch [9,28] including differences in degree of phosphorylation [29] that potentially could influence the efficiency of the esterification process. An interesting observation is that high-amylose maize starch appeared to be the least easily butyrylated after five h of reaction. This is not aligned with previous results [19] and is perhaps partly explained by differences in the routes of esterification.

The solid-state ^13^C MAS NMR analysis revealed that the organocatalytic method involving tartaric acid as a catalyst resulted in much more tartarate (75–89% of the acylation) than butyrate (11–25% of the acylation) added to the starch polymer. This suggests a reaction mechanism involving both acids and it also suggests that results on DS measured by heterogeneous saponification and back titration with HCl (the chemical method) should be confirmed by other methods such as solid-state ^1^^3^C MAS NMR. Another issue is that tartaric acid is a dicarboxylic acid and both of the acid groups may in principle react with a hydroxyl group on a glucose residue in the starch. Therefore, it is possible that the results on DS by tartarate (see Table 2) are overestimated and that the molar ratios between the acid and the anhyroglucose units (also provided in Table 2) are the more correct estimate for DS by tartarate. Tupa et al. [18] from which the present work was inspired did not confirm their findings on DS by the chemical method following esterification with butyrate nor with acetate [30] with alternative methods, but they did, in a study, describe organocatalytic synthesis of propionylated starch [27]. Visual inspection of the ^13^C CP/MAS spectrum of the starch esterified with propionic acid [27] revealed that the integral of CH_2_ as well as CH_3_ were much smaller than the integral of C1 and, therefore, do not correspond to DS = 0.98. In addition, the integral of the carbonyl resonance was much larger (approximately a factor 2) than the integrals of CH_2_ and CH_3_. The excess carbonyl must originate from tartaric acid, which demonstrates that tartaric acid also reacted with starch in their sample preparation. Lopez-Rubio et al. [19] studied the structural modifications of low-amylose and high-amylose maize starch upon acylation with acetate, propionate, and butyrate in aqueous medium with anhydrides and NaOH as a catalyst. They concluded that the longer esterified chains are able to be accommodated within the crystalline lamellar regions so that the starch nanostructure is affected less by butyrylation than by the addition of acetyl or propionyl groups. This makes butyrylated starch less accessible for bacterial hydrolyzing enzymes and less degradable in vitro compared with acetylated and propionylated starch [31]. Overall, it indicates the importance of the acyl group chain length for the nanostructure of the esterified product. Therefore, comparable solid-state ^1^^3^C MAS NMR analysis of acetylated, propionylated, and butyrylated starches produced by the organocatalytic method with tartaric acid should be performed. The characterization of the crystallinity and nanostructure should be used to investigate potential discrepancies in DS depending on the acyl group chain length.

In the current study, the overall rationale behind coupling butyrate to the starch polymer was to make the starch itself more resistant to enzymatic digestion in the small intestine and available for microbial fermentation and colonic SCFA production at the same time as butyrate is delivered directly [14]. An additional question was whether a higher DS with butyrate could increase colonic butyrate delivery and concentration accordingly. In order to look into this, we used an in vitro fermentation model with human fecal inoculum. The chemical method for determination of DS seemed to overestimate the DS compared with the NMR-based method by 38% to 91%. The butyrylated samples used in our in vitro fermentation studies were selected based on their DS and determined by the chemical method. Therefore, the DS shown in Figure 3 and Figure 4 are probably overestimated when considering the NMR results on DS as the “golden standard.” 

Our esterified starch samples were not pre-digested with enzymes, according to the AOAC method (AOAC 991.43) prior to the in vitro fermentation since it is typically practiced in these types of study designs [32]. We only had small amounts (below 500 mg) of esterified starches available and the pre-digestion procedure would not have left enough material to conduct triplicate in vitro fermentations subsequently. No pre-digestion was carried out on the non-treated starches either in order to treat all samples similarly. The presence of starch may have contributed to the relatively high production of total organic acids and butyric acid following fermentation of both non-treated LAMS, HAMS, LAPS, and HAPS. However, the lack of pre-digestion before in vitro fermentation would only have been an actual problem if the esterified starches had resulted in higher productions of organic acids and butyrate than the native starch. In that situation, it would have been difficult to conclude whether the esterified starches were more effective at increasing total organic acid and butyrate production due to the potential presence of starch in the samples. The esterified starches, independent of starch source, appeared to be resistant to microbial fermentation since most of them were not very different from the negative control in both the total organic acid concentration and the butyrate concentration. Only LAPS with a DS = 0.28 resulted in a significantly higher concentration of butyrate compared with the negative control, but this difference was no longer present when calculating the butyrate:total organic acid ratio, which suggests no real increase in butyrate production. Actually, the higher the DS, the more poorly the esterified starches seemed to be fermented by the microbiota. To our knowledge, there are no previous published reports on the in vitro fermentation of butyrylated starches varying in DS. A number of animal studies with butyrylated high-amylose maize starch (DS ≈ 0.25) indicate a high level of butyrate release and fermentation of the residual starch [14,33]. No in vivo results exist on butyrate delivery and fermentation from starches butyrylated to varying DS with methods like the organocatalytic route applied in our study.

## 5. Conclusions

The organocatalytic method can be used for butyrylation of both high amylose and low amylose maize and potato starch to yield products with increasing DS depending on reaction time. The method, however, resulted in much more tartarate than butyrate added to the starch polymer, which suggests a reaction mechanism involving both acids. The chemical method for DS determination highly overestimated the DS compared with solid-state ^13^C MAS NMR. The butyrylated products, independent of a starch source, were only poorly fermented in an *in vitro* fermentation model and butyrylation to high DS with the organocatalytic method does not seem to potentially deliver more butyrate to the large intestine than lower DS products.

## Figures and Tables

**Table 1:** Chemical composition of the low-amylose and high-amylose maize starch (LAMS and HAMS, respectively) and low-amylose and high-amylose potato starch (LAPS and HAPS, respectively).

Chemical Composition (% of DM)	LAMS	HAMS	LAPS	HAPS
DM (%)	91.7	87.8	96.3	86.9
Ash	0.19	0.15	0.34	0.85
Total starch	97.7	97.9 ^4^	97.0	93.7
Amylose ^1^	30.2	79.6	19.3	41.5
Amylopectin ^1^	69.8	20.4	80.7	58.5
RS ^2^	0.8	39.9	0.6	25.3
RS ^3^ (% of total starch)	0.8	40.8	0.6	27.0

^1^ Measured by the Megazyme amylose/amylopectin kit. Amylopectin is calculated from the amylose content. ^2^ Measured by the Megazyme RS kit (Megazyme Internationa, Wicklow, Ireland). ^3^ Calculated from the total starch content determined by the Englyst procedure. ^4^ Determined by direct hydrolysis. Swelling in 12 M H_2_SO_4_ followed by hydrolysis in 2 M H_2_SO_4_ for 1 h and determination of glucose by GC (Gas Chromatography), according to the method described by Bach-Knudsen et al. [26]. DM: dry matter.

**Table 2:** Degree of substitution (DS) determined by solid-state ^13^C MAS NMR spectroscopy for butyrate and tartarate separately compared with the overall DS determined by the chemical method for four selected esterified starches with either low or high DS.

Sample	DS ^1^, Butyrate (NMR)	DS ^1^, Tartarate (NMR)	DS ^1^ (Chemical Method)
HAMS-low DS	0.038	0.31 (31) ^2^	0.35
LAMS-low DS	0.037	0.28 (28) ^2^	0.44
HAPS-low DS	0.022	0.08 (8) ^2^	0.17
LAPS-low DS	0.035	0.16 (16) ^2^	0.30
HAMS-high DS	0.11	0.92 (92) ^2^	1.49
LAMS-high DS	0.23	1.16 (116) ^2^	2.48
HAPS-high DS	0.27	0.92 (92) ^2^	2.27
LAPS-high DS	0.31	0.93 (93) ^2^	2.26

^1^ DS: the number of OH-groups substituted by an acyl group per anhydroglucose unit of the starch polymer. Since the anhydroglucose unit possesses three reactive OH-groups, the maximum DS value is 3. ^2^ Numbers in brackets refers to DS with tartarate in mol% in relation to glucose monomers in the starch, i.e., mol%= 31: on average 1 tartarate is attached to 31% of the glucose monomers in starch.

## Founding

The study was financially supported by the Independent Research Fund Denmark in the project “Starch based nutraceuticals—can starch improve colonic health?” (project No. 4005-00258).
